# Supplementary material for: Light-induced manipulation of passive and active microparticles
Source: Eur Phys J E Soft Matter. 2021 Apr 8;44(4):50. doi: 10.1140/epje/s10189-021-00032-x (PMC8032649; doi:10.1140/epje/s10189-021-00032-x)
Supplement: Supplementary file 3 — Supplementary material 3 (pdf 49 KB) [file 10189_2021_32_MOESM3_ESM.pdf]

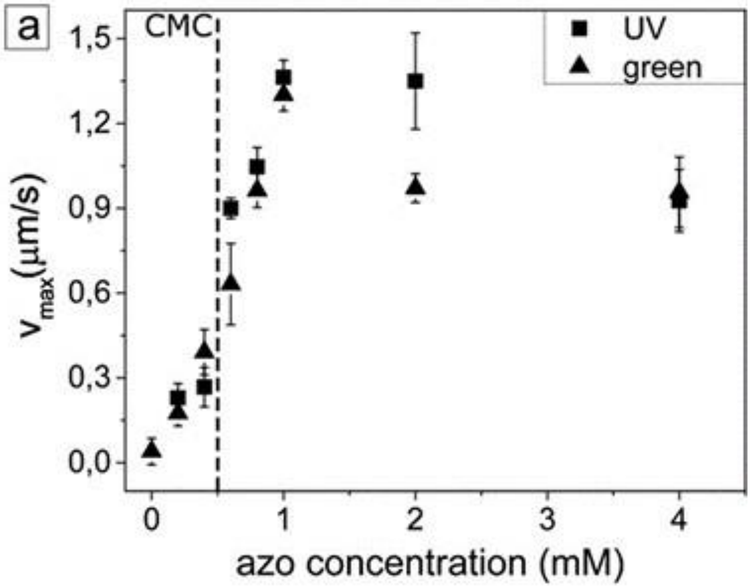

Figure S3. Maximum velocity of 2 µm nonporous silica particles for different surfactant concentration under focused UV and green irradiation. Plot is taken from Scientific Report 6, 36443 (2016).
